# The Susceptibility of Two *Beauveria bassiana* Strains on Rice Pests *Nilaparvata lugens* and *Sogatella furcifera*

**DOI:** 10.3390/jof11020128

**Published:** 2025-02-08

**Authors:** Zhongwei Chen, Hanqing Mu, Yifan Peng, Rui Huo, Jiaqin Xie

**Affiliations:** 1 School of Life Sciences, Genetic Engineering Research Center, Chongqing University, Chongqing 400044, China; 202326131053@stu.cqu.edu.cn (Z.C.); mhq19900828@163.com (H.M.); 2 Wuhan Kernel Bio-tech Co., Ltd., Guannanyuan Road No.17, Guannan Industrial Park, Wuhan 430074, China; pyifan@yeah.net (Y.P.); 597568519@163.com (R.H.); 3 National Engineering Research Center of Microbial Pesticides (Joint Institute-Chongqing University) and Chongqing Engineering Research Center for Fungal Insecticides, Chongqing 400044, China; 4 Key Laboratory of Gene Function and Regulation Technology Under Chongqing Municipal Education Commission, Chongqing 400044, China

**Keywords:** entomopathogenic fungi, biological agent, rice planthopper, pest control

## Abstract

Entomopathogenic fungi represent a valuable natural resource with significant potential as biological agents for pest management. However, different species or strains of fungi demonstrate varying effectiveness against specific targets. In this study, we assessed the impact of two fungal strains, *Beauveria bassiana* KN801 and KN802, on the rice planthoppers *Ninaparvata lugens* and *Sogatella furcifera*, in combination with insecticides. Our findings indicate that both *B. bassiana* strains can effectively infect the nymphs and adults of *N. lugens* and *S. furcifera*, resulting in a significantly higher mortality rate compared to the control groups. Notably, the *B. bassiana* strain KN801 demonstrated greater virulence than *B. bassiana* KN802 against these pests. However, no significant differences were observed when using different concentrations of the same fungal strain (*B. bassiana* KN801 or *B. bassiana* KN802) against these targets. Additionally, both fungi showed a germination rate of over 90% after treatment when combined with several common insecticides like chlorfenapyr and dinotefuran. The combined application of *B. bassiana* with chlorfenapyr or dinotefuran could improve pest control efficacy for these two pests. This study suggests that the two *B. bassiana* strains have the potential to infect rice planthoppers *N. lugens* and *S. furcifera*, indicating their promise as agents for the control of these pests.

## 1. Introduction

The rice planthopper is a key pest for rice plants, causing significant damage by directly feeding on the sap of rice plants [1], and also by transmitting diseases such as *the rice ragged stunt virus*, *the rice grassy stunt virus*, and *the south rice black-streaked dwarf virus* as vectors [2,3]. Traditional control methods, primarily chemical insecticides, have proven unsustainable for crop development [4]. Misuse of chemical insecticides has led to numerous environmental and nontarget organism side effects, including the decline of pollinator populations and disruption of natural enemy behavior [5,6]. Additionally, these insect pests have developed high resistance to commonly used chemicals such as imidacloprid and pymetrozine [7]. Therefore, the search for more effective and environmentally friendly approaches or alternatives for controlling these pests is imperative.

Entomopathogenic fungi (EF) are abundant in nature and play crucial roles in regulating insect populations. Certain EF strains have been reported to be effective in infecting insect pests, such as *Diaphorina citri* [8] and *Busoniomimus manjunathi* [9]. However, it is important to note that different fungal species or strains exhibit varying effects on targeting insect hosts, suggesting the need for specific fungal strains to effectively infect and control particular insect pests. In addition to their impact on insects, EF have also been reported to have beneficial effects on plants, yielding positive ecological outcomes [10]. Some EF can enhance host plants’ nutrient intake, resistance to pathogens, and promote growth [11]. Several studies have demonstrated that EF improve host plant utilization of nitrogen and carbon via the endosphere [12,13] while also enhancing resistance to insect pests [14]. These findings underscore the potential of EF as a valuable resource for sustainable pest management and plant health.

EF possess a unique ability to directly penetrate the cuticle of insect hosts through complex processes [15]. Once inside the host’s body, they release toxins that harm and weaken the host’s immune response, facilitating their infection [16]. Subsequently, the EF acquire nutrients from the host’s body for their own growth, ultimately leading to the death of the insect host. The penetration strategy of entomopathogenic fungi makes it challenging for insect hosts to develop resistance to fungal infection [17]. As a result, certain fungal strains have been employed to control resistant insects, and engineered fungal strains have the potential to enhance their performance against such pests [18,19]. In contrast to chemical approaches, EF present fewer risks to the environment and may have sustained effects on insect populations, due to their survival traits in soil or water [11,20]. Therefore, the utilization of EF holds significant promise as an alternative to insecticides for the control of insect pests.

The outbreak of pest populations in a specific area, due to climate change or human activity, can cause significant damage to crops within a short period. While EF can be utilized to target and control these pests, it often requires some time to effectively reduce their numbers and suppress the population to a low level. Certain fungal strains may show limited control potential when used alone [10]. In such scenarios, integrated pest management (IPM) or the combined use of EF and other control approaches becomes a favorable option [11,21]. In practice, some successful applications have involved the combined use of fungi and insecticides for controlling insect pests, yielding positive results [22]. When EF poses a risk (albeit lower than that associated with chemical pesticides) to beneficial insects or natural enemies of the target pest, significant biological control effects can still be achieved by staggering the application times of the two agents [23]. Consequently, the careful and strategic evaluation of EF in conjunction with insecticides can provide a more effective approach for the control of insect pests.

In this study, we examined the impact of two different strains of *B. bassiana* on different stages of significant rice pests, specifically *N. lugens* and *S. furcifera*. Furthermore, we evaluated the compatibility of these two strains with 10 commonly used insecticides for rice pest management. Our investigation focused on the synergistic application of these fungi with low dosages of insecticides to combat rice planthoppers. The outcomes of this research may introduce novel *B. bassiana* strains for effectively managing rice planthoppers, providing alternative approaches to rice pest control.

## 2. Materials and Methods

### 2.1. Insects and B. bassiana Strains

The rice planthoppers *N. lugens* and *S. furcifera* utilized in this study were sourced from laboratory populations in the insectary room at Chongqing University, Chongqing, China. All insects were maintained on rice seedlings within plastic cages (30 cm × 30 cm × 40 cm) under ambient conditions, with a temperature of 26 ± 1 °C and a light cycle of 14:10 h (Dark: Light). Prior to the commencement of the experiments, female and male rice planthoppers *N. lugens* or *S. furcifera* were conducted into new cages with fresh rice seedlings. After 48 h, all individuals were removed from the cages following egg laying. Subsequently, five-instar nymphs and newly emerged *N. lugens* and *S. furcifera* were collected for use in this experiment. The conditions of the rice plants were consistently monitored throughout the experiments, ensuring they were watered daily to maintain their freshness. This meticulous care and maintenance of the experimental setup were crucial to ensuring the health and viability of the rice planthoppers and the accuracy of the study’s results.

The entomopathogenic fungal strains *B. bassiana* KN801 and KN802 were obtained from Wuhan Kernel Bio-tech Co. in Wuhan, China. Strain *B. bassiana* KN801 (Identification No. 20231211-1) and KN802 (Identification No. 20231211-2) were recently isolated from aphids in Wuhan and *Spodoptera frugiperda* in Yunnan, respectively. These strains have been identified and characterized by the China Microbial Culture Preservation Management Committee (Agricultural Microbiology Center, Beijin, China). To conduct bioassay evaluations, *B. bassiana* KN801 and KN802 were initially inoculated onto SDAY medium (containing 10 g glucose, 5 g yeast extract, 2.5 g peptone, and 18 g agar per liter of sterilized water) by streaking on a Petri dish. These plates were then placed in a constant temperature incubator set at 27 °C for a period of two weeks. Once the spores reached maturity, an inoculation loop was used to collect an appropriate quantity of spores, which were subsequently transferred to a 0.05% Tween-80 solution.

The spore-Tween-80 mixture was then vigorously shaken and mixed for three to five minutes using a vortex oscillator. Following this, the mixed solution was filtered through pre-sterilized four-layer lens paper to eliminate impurities such as mycelium and medium, resulting in the original spore suspension. This original suspension was then gradient-diluted with 0.05% Tween-80 solution to obtain spore suspensions at concentrations of 1 × 10^5^, 1 × 10^6^, 1 × 10^7^, and 1 × 10^8^ conidia per milliliter for use in further experiments.

### 2.2. Bioassay Analysis of B. bassiana to N. lugens and S. furcifera

To investigate the potential impacts of *B. bassiana* strains KN801 and KN802 on rice pests *N. lugens* and *S. furcifera*, the survival of these pests was evaluated after being exposed to various concentrations of *B. bassiana*. A plastic cup measuring with dimensions of 9 cm in diameter and 15 cm in height, containing fresh rice seedlings, was used for this experiment. Twenty individuals of *N. lugens* or *S. furcifera*, including five-instar nymphs and adults, were collected and transferred into each cup. The cups were then covered with gauze to facilitate air circulation and to prevent the planthoppers from escaping.

Following the transfer, a Potter Precision Spray Tower manufactured by Burkard, Hertfordshire, UK, was utilized to spray 500 μL of each concentration (i.e., 1 × 10^5^, 1 × 10^6^, 1 × 10^7^, and 1 × 10^8^ conidia/mL) of *B. bassiana* KN801 or KN802 suspension into the cups. The control group was sprayed with an equal volume of 0.05% Tween-80 solution. The spraying operation pressure was set at 0.5 Bar. Subsequently, the plastic cups were transferred to a biotest chamber for cultivation, maintaining constant environmental conditions including a temperature of 26 ± 1 °C, a light cycle of 14 h of light and 10 h of dark, and humidity within the range of 70 ± 5%. Each treatment was conducted with three replicates. Throughout the experiment, the survival of *N. lugens* and *S. furcifera* in each treatment cup was monitored, and fresh water was provided daily up to the 8th days. The LT_50_ of each treatment was determined through Probit analysis using GraphPad Prism 9 and further analyzed with Tukey’s test with the control treatment. This approach allowed us to observe and assess the impact of *B. bassiana* strains on the survival of the rice pests in a controlled environment.

### 2.3. Compatibility Analysis of B. bassiana with Insecticides

In our further assessment, we aimed to determine the compatibility of *B. bassiana* strains KN801 and KN802 with ten commonly used insecticides for the control of rice pests (refer to Table 1). These insecticides were prepared at twice the recommended dosages as specified by the producers. Subsequently, a spore suspension of *B. bassiana* KN801 and KN802 at a concentration of 2 × 10^7^ conidia/mL was prepared utilizing the method previously described.

The spore suspension was then combined with each insecticide in a 1:1 ratio to create a compound liquid. This compound liquid was thoroughly mixed using a vortex oscillator for 3 to 5 min after the components were added together. Following this initial mixing, the mixture was allowed to stand at room temperature for 4 h, with the compound liquid being shaken again using the vortex oscillator every hour. Finally, 100 μL of the compound liquid was spread on SDAY medium and placed in a constant temperature incubator at 27 °C. We observed the samples at 6 and 12 h to determine the germination rate under an optical microscope (germination rate = number of germinated spores/total number of spores, with spore germination being determined by the emergence of germ tubes). For each sample, 100 individual conidia were counted, and three replicates were conducted for each treatment. Subsequently, a *t*-test was performed to analyze the compatibility of chemical insecticides with the two fungus strains. This rigorous approach allowed us to assess the impact of the insecticides on the germination of *B. bassiana* spores, providing valuable insights into their compatibility.

### 2.4. Effects of the Combined Use of B. bassiana with Insecticides on Rice Planthopper

In our investigation of the combined effects of fungi and insecticides, we have selected chlorfenapyr and dinotefuran as the target insecticides for testing with *B. bassiana* strains KN801 and KN802, respectively, based on their compatibility with the fungi. To begin, 26.5 μL of the chlorfenapyr formulation was added to 10 mL of 0.05% Tween-80 solution to create a 2× solution. Subsequently, this was further diluted to 20% and 40%, and then mixed with a 2 × 10^7^ conidia/mL *B. bassiana* KN801 spore suspension at a 1:1 ratio. The solution was thoroughly mixed using a vortex oscillator for 3 to 5 min, and then allowed to stand at room temperature for 4 h, with intermittent shaking using the vortex oscillator every hour.

After the preparation, *N. lugens* and *S. furcifera* adults were treated according to the bioassay method outlined previously. Each treatment was conducted with three replicates, and the daily number of surviving *N. lugens* and *S. furcifera* individuals was recorded. The approach allowed us to assess the impact of the combined application of the selected insecticides and *B. bassiana* strains on the survival of these target insects, providing insights into the potential synergistic or antagonistic effects of the combined treatments.

In the investigation of the combined effects of dinotefuran and *B. bassiana* KN802, a total of 0.173 g of the dinotefuran formulation was dissolved in 10 mL of 0.05% Tween-80 solution to create a 2× solution. This solution was then further diluted to 6% and 10% and mixed with a 2 × 10^7^ conidia/mL *B. bassiana* KN802 spore suspension at a 1:1 ratio. The resulting mixture was thoroughly agitated using a vortex oscillator for three to five minutes and then allowed to stand at room temperature for four hours, with intermittent shaking using the vortex oscillator every hour. Following this preparation, a group of 20 *N. lugens* and *S. furcifera* adults were treated according to the bioassay method outlined previously. Each treatment was conducted with three replicates, and the daily number of surviving *N. lugens* and *S. furcifera* individuals was recorded up to the eighth day. The LT_50_ and LT_90_ of each treatment were determined through Probit analysis with χ^2^ using GraphPad Prism 9 and further analyzed using Tukey’s test with the control treatment. The approach enabled us to assess the potential combined impact of dinotefuran and *B. bassiana* KN802 on the survival of these target insects, providing insights into the interactions between the insecticide and the fungal strain.

### 2.5. Statistical Analysis

To analyze the data, the normality of the data and the homogeneity of variances were assessed using the Shapiro–Wilk and Levene tests, respectively. The effects of different concentrations of KN801 and KN802 on *N. lugens* and *S. furcifera* individuals were analyzed using one-way analysis of variance (ANOVA) followed by Tukey’s test. The compatibility was examined by *t*-test, while combined use were also analyzed by ANOVA post with Tukey’s test. Subsequently, the results of the LT_50_ or LT_90_ were then subjected to Probit analysis with χ^2^ using GraphPad Prism 9, followed by further analysis using Tukey’s test. A significance level of *p* < 0.05 was applied to determine statistical significance, ensuring that any observed differences were not due to random variation.

## 3. Results

### 3.1. Susceptibility of B. bassiana to S. furcifera

**Figure 1:**
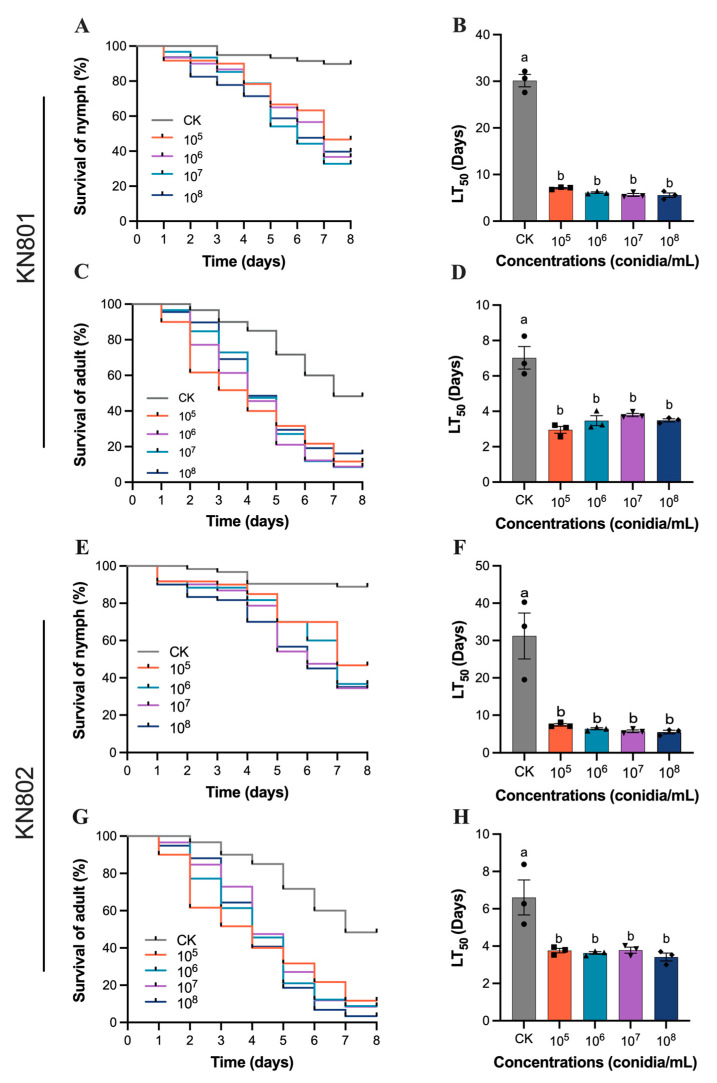
The susceptibility evaluation of *B. bassiana* strains KN801 and KN802 to *S. furcifera*. (**A**,**B**) the survival rate and LT_50_ of *B. bassiana* strain KN801 to *S. furcifera* nymph. (**C**,**D)** the survival rate and LT_50_ of *B. bassiana* strain KN801 to *S. furcifera* adult. (**E**,**F**) the survival rate and LT_50_ of *B. bassiana* KN802 to *S. furcifera* nymph. (**G**,**H**) the survival rate and LT_50_ of *B. bassiana* KN802 to *S. furcifera* adult. The error bars indicate Standard error (SE) in the graphs. Different letters indicate a significant difference with *p* < 0.05.

This study focused on assessing the efficacy of the fungal *B. bassiana* against the rice pests *N. lugens* and *S. furcifera*, specifically targeting both the fifth instar nymphs and adults of *S. furcifera*. It was observed that *B. bassiana* strains KN801 and KN802 demonstrated effective control against the targeted pests. Following treatment with *B. bassiana* KN801 at a concentration of 1 × 10^5^ conidia/mL, the LT_50_ values were 7.08 ± 0.27 days (F_4,10_ = 266.6, *p* < 0.001; Figure 1A,B) for nymphs and 2.96 ± 0.34 days (F_4,10_ = 24.86, *p* < 0.001; Figure 1C,D) for adults. Similarly, treatment with *B. bassiana* KN802 at the same concentration resulted in LT_50_ values of 5.02 ± 0.27 days (F_4,10_ = 16.43, *p* = 0.0002; Figure 1E,F) for nymphs and 4.08 ± 1.42 days (F_4,10_ = 9.113, *p* = 0.0023; Figure 1G,H) for adults. These values were significantly lower than those of the control group, indicating the substantial impact of the fungal strains on pest mortality. Furthermore, it was noted that as the concentration of the spore suspension increased, there was no significant difference in LT_50_ values between the nymphs and adults of *S. furcifera* for both *B. bassiana* KN801 and KN802.

### 3.2. Susceptibility of B. bassiana to N. lugens

**Figure 2:**
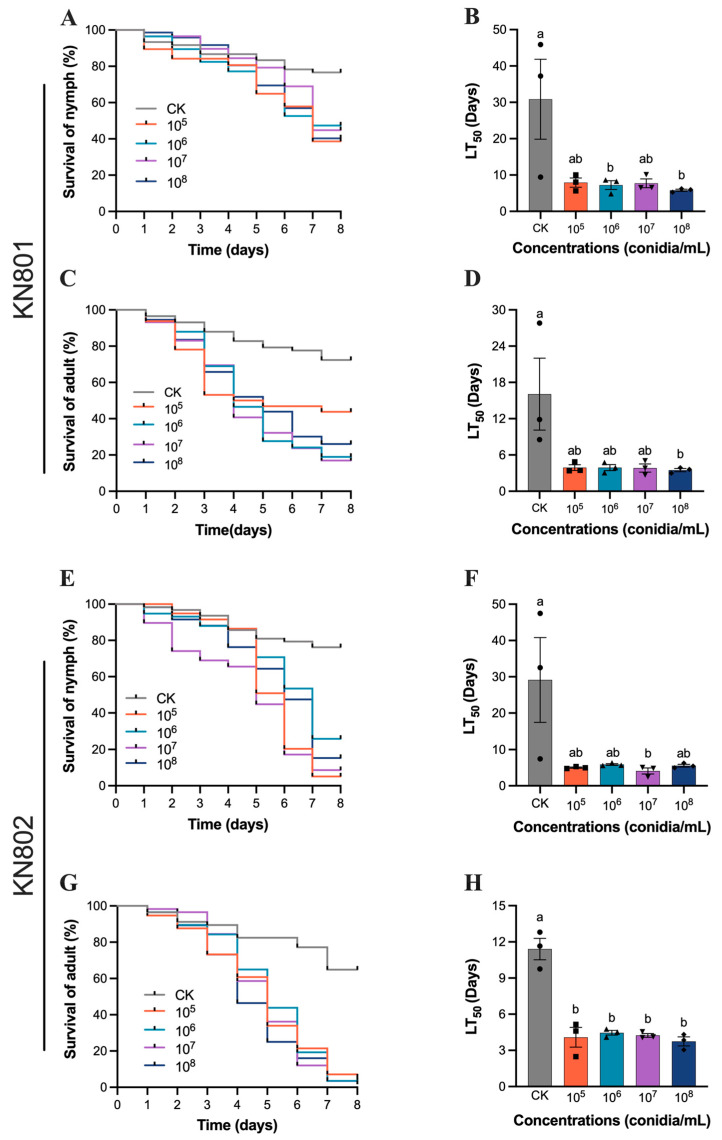
The susceptibility evaluation of *B. bassiana* strains KN801 and KN802 to *N. lugens*. (**A**,**B**) the survival rate and LT_50_ of *B. bassiana* KN801 to *N. lugens* nymph. (**C**,**D**) the survival rate and LT_50_ of *B. bassiana* KN801 to *N. lugens* adult. (**E**,**F**) the survival rate and LT_50_ of *B. bassiana* KN802 to *N. lugens* nymph. (**G**,**H**) the survival rate and LT_50_ of *B. bassiana* KN802 to *N. lugens* adult. The error bars indicate the Standard error (SE) in the graphs. Different letters indicate a significant difference with *p* < 0.05.

The study also demonstrated the effectiveness of *B. bassiana* strains KN801 and KN802 in controlling the fifth instar nymphs and adults of the rice planthopper *N. lugens*. After treatment with *B. bassiana* KN801 at a concentration of 1 × 10^5^ conidia/mL, the LT_50_ values were 7.92 ± 2.2 days (F_4,10_ = 4.496, *p* = 0.0245; Figure 2A,B) for nymphs and 3.91 ± 0.82 days (F_4,10_ = 4.149, *p* = 0.0310; Figure 2C,D) for adults. Similarly, treatment with *B. bassiana* KN802 at the same concentration resulted in LT_50_ values of 5.02 ± 0.27 days (F_4,10_ = 4.205, *p* = 0.0298; Figure 2E,F) for nymphs and 4.08 ± 1.42 days (F_4,10_ = 32.16, *p* < 0.0001; Figure 2G,H) for adults. These values were lower than those of the control group, indicating the significant impact of the fungal strains on pest mortality.

In addition, for *B. bassiana* KN801, spore suspensions at 1 × 10^6^ and 1 × 10^8^ conidia/mL showed significant lethal effects on *N. lugens* nymphs. The 1 × 10^8^ conidia/mL suspension exhibited a significant lethal effect on brown planthopper adults (*p* = 0.0495, Tukey’s test). Similarly, for *B. bassiana* KN802, the 1 × 10^7^ conidia/mL spore suspension showed a significant lethal effect on nymphs (*p* = 0.0438, Tukey’s test), while all concentrations showed a significant lethal effect on adults (all *p* < 0.0001, Tukey’s test). These results highlight the potential of *B. bassiana* strains KN801 and KN802 as effective agents for controlling *N. lugens* populations, particularly in targeting both nymphs and adults.

### 3.3. Compatibility of B. bassiana Strains with Insecticides

**Figure 3:**
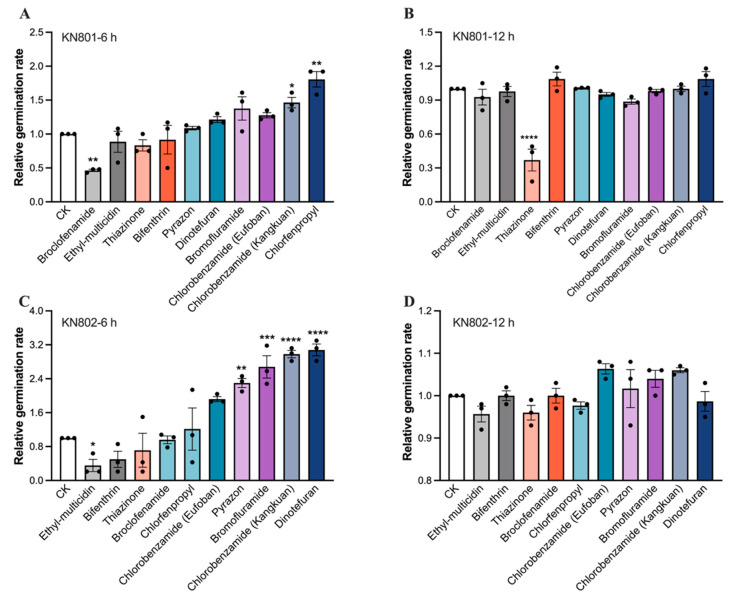
The compatibility of *B. bassiana* strains KN801 and KN802 with insecticides. (**A**,**B**) the relative germination of *B. bassiana* KN801 mixed with insecticides at 6 and 12 h. (**C**,**D**) the relative germination of *B. bassiana* KN802 mixed with insecticides at 6 and 12 h. The error bars indicate the Standard error (SE) in the graphs. An asterisk “*” indicates a significant difference between two treatments with *p* < 0.05, “**” indicates a significant difference between two treatments with *p* < 0.01, “***” indicates a significant difference between two treatments with *p* < 0.001, “****” indicates a significant difference between two treatments with *p* < 0.0001.

The study also evaluated the compatibility of *B. bassiana* strains KN801 and KN802 with ten commonly used insecticides for rice pests. The results revealed that the germination of *B. bassiana* KN801 was significantly inhibited by the insecticide bromochloronicotinyl at 6 h (*p* = 0.0145, Figure 3A; *t*-test), with only half the germination rate of the control group. However, chlorfenapyr and cyflumetofen did not have a significant effect on spore germination for KN801 (chlorfenapyr, *p* = 0.0420; cyflumetofen, *p* = 0.0003; Figure 3A). At 12 h, only thiacloprid exhibited significant inhibition, with a germination rate only one-third of the control group (*p* < 0.0001, Figure 3B).

For *B. bassiana* strain KN802, the spore germination rates in the groups treated with ethaboxam, deltamethrin, and thiacloprid were lower than the control group but had no significant effect at 6 h (Figure 3C). Conversely, acetamiprid, flonicamid, chlorfenapyr, and flubendiamide promoted spore germination of *B. bassiana* KN802, with germination rates about three times that of the control group (acetamiprid, *p* = 0.0050; flonicamid, *p* = 0.0003; chlorfenapyr_convict, *p* < 0.0001; flubendiamide, *p* < 0.0001; Figure 3C). However, the other tested insecticides had no significant effect on *B. bassiana* germination. Furthermore, all insecticides had no significant effect on KN802 germination after 12 h (Figure 3D). These findings underscore the differential effects of various insecticides on the germination of *B. bassiana* strains KN801 and KN802.

### 3.4. Effects of the Combined Used for N. lugens and S. furcifera

**Figure 4:**
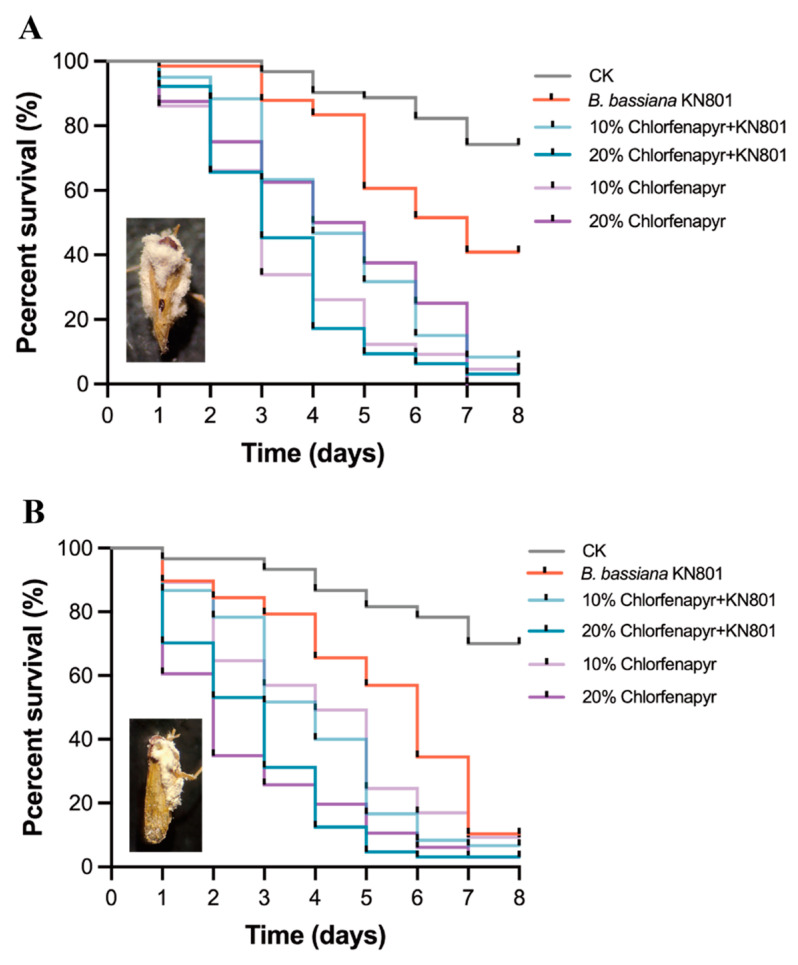
Effects of combined use of *B. bassiana* KN801 and insecticide chorfenapyr for *S. furcifera* and *N. lugens* adults. (**A**) the survival of *S. furcifera* after treating with combined use of *B. bassiana* KN801 and chlorfenapyr. (**B**) the survival of *N. lugens* after treating with combined use of *B. bassiana* KN801 and chlorfenapyr.

Based on the results provided, it appears that the effects of *B. bassiana* and the insecticides chlorfenapyr and dinotefuran were investigated on *N. lugens* and *S. furcifera*. When *S. furcifera* was treated with low concentrations of chlorfenapyr (10% and 20% of the recommended dosage) in combination with *B. bassiana*, it led to significantly shorter LT_50_ compared to using *B. bassiana* KN801 alone, regardless of whether the concentration was at 10% or 20%. However, the LT_90_ for the combined use at 20% chlorfenapyr was significantly lower than the use of *B. bassiana* alone, whereas 10% chlorfenapyr showed no significant difference in LT_90_ (χ^2^ = 165.2, *p* < 0.001; Table 2 and Figure 4A).

**Figure 5:**
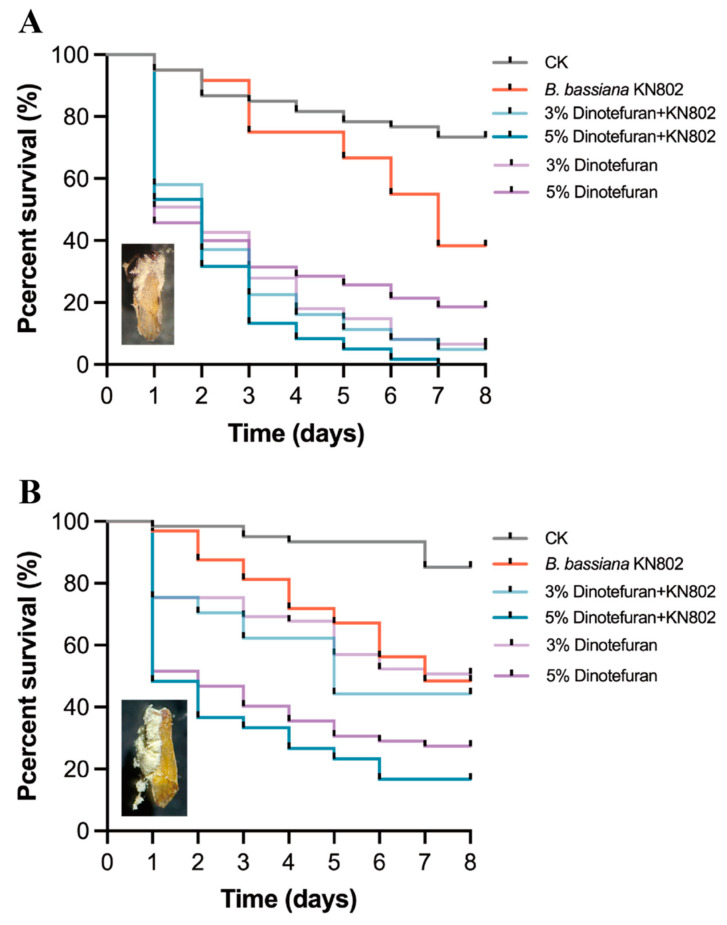
Effects of combined use of *B. bassiana* KN802 and insecticide dinotefuran for *S. furcifera* and *N. lugens* adults. (**A**) the survival of *S. furcifera* after treating with combined use of *B. bassiana* KN802 and dinotefuran. (**B**) the survival of *N. lugens* after treating with combined use of *B. bassiana* KN802 and dinotefuran.

When testing on *N. lugens*, using 10% chlorfenapyr showed no significant difference compared to using *B. bassiana* KN801 alone. However, when using 20% chlorfenapyr, the LT_50_ and LT_90_ for the combined use were significantly shorter than using abamectin alone (χ^2^ = 154.6, *p* < 0.001; Table 2 and Figure 4B). Similarly, when using *B. bassiana* KN802 in combination with 3% or 5% of dinotefuran (χ^2^ = 148.9, *p* < 001; Table 3 and Figure 5A), it resulted in significantly shorter LT_50_ and LT_90_ compared to using *B. bassiana* KN802 alone for *S. furcifera*. However, when considering *N. lugens* (Table 3 and Figure 5B), using 3% dinotefuran alone showed no significant difference compared to using *B. bassiana* KN802 alone. Nevertheless, when using 5% dinotefuran, the LT_50_ for the combined use was significantly shorter than using dinotefuran alone, while the LT_90_ showed no significant difference (χ^2^ = 85.82, *p* < 0.001; Table 3 and Figure 5B).

## 4. Discussion

Entomopathogenic fungi offer a safer and more effective alternative to commonly used insecticides for managing pests and pathogens [14,24]. However, it is important to note that the efficacy and specificity of EF (i.e., whether they target a wide range of insects or specific species) can vary, even within the same insect species [25,26]. Therefore, careful consideration of the specific fungi that target particular insect pests is crucial before implementing them for pest control. In this study, the focus was on evaluating the effects of two new strains of the fungal species *B. bassiana* (strains KN801 and KN802) on a major rice pest. Additionally, the study examined the susceptibility of these fungi to several insecticides. The results indicated that *B. bassiana* can infect and cause the death of the rice planthopper, and the combined use of these fungi and certain insecticides may enhance their efficiency, thereby presenting a promising approach for rice pest control. Their unique mode of action and potential for sustained effectiveness make EF a compelling option for pest management, particularly in light of the challenges posed by insecticide resistance and environmental concerns associated with chemical insecticides.

The rice planthopper is a persistent pest for rice plants, and has been known to cause substantial damage. Numerous studies have documented that populations of rice planthoppers have developed high resistance to many insecticides, including pyrethroids and imidacloprid [7,27]. Insects have evolved to resist these chemicals by modifying the channels or targets that the insecticides act upon [28]. This resistance has led to a situation where new or more potent insecticides are being used to combat this rice pest, often with high environmental risks and increased costs associated with their application. Importantly, there is a growing need to reduce the reliance on chemical insecticides and seek safer alternatives [29]. This demand has prompted the search for effective approaches or products for pest control, and entomopathogenic fungi emerge as a promising choice due to their inherent traits.

While some entomopathogenic fungi, such as *Cladosporium* sp., *M. indicum*, and *B. bassiana*, have been utilized for the control of various pests, there remains a wealth of untapped resources in the form of more effective fungi [30,31,32]. Only a few entomopathogenic fungi have been specifically studied and reported as useful for controlling rice pests in field applications [26,33]. This study contributes to the evaluation of two new *B. bassiana* strains for the control of the rice planthopper. The findings of this study revealed that the two *B. bassiana* strains exhibited differing pathogenic effects, with different lethal efficiencies (i.e., LT_50_ = 4.84 vs. 6.72 for *N. lugens* and 5.87 vs. 6.15 for *S. furcifera*) on the rice planthopper. This indicates the variable functionality of entomopathogenic fungi with respect to specific insect hosts. Similarly, other studies have reported that different fungal strains, such as *M. anisopliae* Ma41 and *M. anisopliae* Ma22, demonstrated varying functions [34]. As a result, it is crucial to thoroughly assess the potential of fungal strains for specific hosts before considering their further use or commercialized development. However, it was noted that the different concentrations of *B. bassiana* showed no significant difference from two tested rice planthoppers. This lack of differentiation could potentially be attributed to a loading threshold on the insect body achieved by spraying methods with a similar volume. Further examination is required for a more precise assessment in this regard.

The complex nature of the process by which fungi act on insect hosts implies that it may take a longer time to induce the death of insect pests compared to the immediate action of insecticides [35,36,37]. This prolonged process may limit the efficiency of entomopathogenic fungi, especially when facing rapid pest outbreaks. Additionally, the cost associated with using entomopathogenic fungi for pest control can be relatively high due to the extended timeframe [38]. Several studies have demonstrated that the combined use of fungi and insecticides can be an effective approach for pest control [39,40]. However, the success of employing a combined approach hinges on the susceptibility of different fungi strains to various insecticides, a factor that can vary even among the same type of insecticide produced by different manufacturers [41]. Different chemical insecticides contain distinct components that target specific areas within the insect body. Therefore, it is essential to evaluate the susceptibility of specific fungi strains, such as *B. bassiana* KN801 and KN802, to a range of commonly used insecticides for rice pest control. In our evaluation, we noted variability in the susceptibility of *B. bassiana* strains to these insecticides, highlighting the importance of thoughtful selection when combining insecticides with entomopathogenic fungi. Furthermore, studies to investigate the underlying mechanisms behind the diverse outcomes observed with different insecticide-fungus combinations would be an intriguing avenue for future research. Understanding these mechanisms can provide valuable insights for optimizing pest management strategies and enhancing the efficacy of integrated pest control approaches.

In the combined use of EF and insecticides, it is important to note that using a lower dosage of insecticides, as per the recommended guidelines, may potentially reduce the side effects on the environment. Furthermore, this approach can weaken the target pests and even enhance the effectiveness of the entomopathogenic fungi. Our study has specifically evaluated the combined effects of *B. bassiana* strains KN801 and KN802 with the insecticides chlorfenapyr and dinotefuran, demonstrating that this combination indeed improves the efficiency of *B. bassiana* in controlling rice planthoppers. The combined use of these fungi with specific insecticides has been shown to significantly shorten the time required to induce the death of the target rice planthopper. In practical applications, this combination approach offers the dual benefits of pest prevention and reduction in the workload required for pest control.

## 5. Conclusions

This study focused on evaluating two identified *B. bassiana* strains KN801 and KN802, for their effectiveness in controlling major rice planthopper pests. The results revealed that *B. bassiana* KN801 exhibited a superior performance compared to KN802 in terms of different LT_50_ values for the targeted pests. Moreover, the susceptibility of both strains to various insecticides suggested that *B. bassiana* KN801 combined with chlorfenapyr and *B. bassiana* KN802 combined with dinotefuran could serve as promising approaches for practical use. These findings suggest that the newly identified *B. bassiana* strains hold promise for integrated pest management strategies and could contribute to more effective and sustainable control of rice pests in agricultural settings.

## Figures and Tables

**Table 1:** The ten common insecticides were used for the compatibility test with *B. bassiana*.

Insecticides	Concentrations/Dosages	Producers
Dinotefuran	865 μg/mL	Tongzhou Zhongda Agrochemical Co., Ltd., Nantong, China
Thiazinone	0.337 5 μL/mL	Shenzhen Noposion Agrochemicals Co., Ltd., Shenzhen, China
Bifenthrin	0.165 μL/mL	Yangnong Chemical, Yangzhou, China
Pyrazon	5.325 mg/mL	Syngenta Nantong Crop Protection Co., Ltd., Nantong, China
Chlorobenzamide (Eufoban)	1.1725 mg/mL	Bayer AG, Leverkusen, Germany
Ethyl-multicidin	120 μg/mL	Dow AgroSciences China Limited, Nantong, China
Bromofluramide	0.1 μL/mL	Mitsui Chemicals, Inc., Shanghai, China
Broclofenamide	0.2 μL/mL	Hebei Longping Agricultural Science and Technology Co., Ltd., Shijiazhuang, China
Chlorobenzamide (Kangkuan)	133.4 μg/mL	DuPont, Wilmington, DE, USA
Chlorfenpropyl	0.265 μL/mL	BASF Plant Protection Limited Company, Nantong, China

**Table 2:** Effects of the combined use of *B. bassiana* strain KN801 and insecticide chlorfenapyr for *S. furcifera* and *N. lugens*.

Lethal Time	Targets	*B. bassiana*	10% Chlorfenapyr + *B. bassiana*	20% Chlorfenapyr + *B. bassiana*	10% Chlorfenapyr	20% Chlorfenapyr	CK
LT_50_ with 95% CIs (days)	*N. lugens*	4.84 (4.46~5.22)	3.4 (2.6~4.2)	2.28 (2.11~2.45)	3.99 (3.75~4.23)	1.76 (1~2.52)	8.87 (8.37~9.38)
	*S. furcifera*	5.87 (5.34~6.4)	4.02 (3.97~4.06)	2.89 (2.31~3.47)	2.9 (2.79~3.01)	2.63 (2.34~2.92)	8.89 (7.56~10.21)
LT_90_ with 95% CIs (days)	*N. lugens*	7.88 (6.87~8.89)	5.7 (3.94~7.45)	4.48 (4.14~4.83)	6.75 (5.84~7.65)	3.9 (2.33~5.47)	13.92 (12.9~14.93)
	*S. furcifera*	8.83 (8.47~9.19)	6.44 (5.63~7.26)	4.76 (3.54~5.97)	5.38 (5.14~5.61)	4.56 (4.19~4.94)	12.73 (10.85~14.61)

**Table 3:** Effects of the combined use of *B. bassiana* strain KN802 and insecticide dinotefuran for *S. furcifera* and *N. lugens*.

Lethal Time	Targets	*B. bassiana*	3% Dinotefuran + *B. bassiana*	5% Dinotefuran + B. *bassiana*	3% Dinotefuran	5% Dinotefuran	CK
LT_50_ with 95% Cis (days)	*N. lugens*	6.72 (7.66~5.78)	5.23 (6.05~4.41)	2.18 (2.91~1.44)	6.42 (7.74~5.11)	3.01 (4.3~1.73)	10.83 (13~8.66)
	*S. furcifera*	6.15 (6.43~5.86)	2.07 (2.96~1.18)	1.61 (1.78~1.43)	2.02 (2.1~1.95)	1.61 (2.48~0.74)	9.77 (10.35~9.2)
LT_90_ with 95% Cis (days)	*N. lugens*	11.4 (14.16~8.64)	11.09 (11.78~10.41)	6.74 (8.13~5.34)	13.28 (15.09~11.47)	8.71 (11.24~6.19)	15.68 (20.58~10.78)
	*S. furcifera*	10.18 (11.02~9.34)	4.63 (6.82~2.45)	3.57 (4.27~2.88)	5.46 (6.02~4.9)	5.46 (6.19~4.72)	17.07 (17.68~16.46)

## Data Availability

Data will be made available on request.
